# Predictor of Early Remission of Diabetic Macular Edema under As-Needed Intravitreal Ranibizumab

**DOI:** 10.1038/s41598-019-44078-6

**Published:** 2019-05-20

**Authors:** Tatsuya Yoshitake, Tomoaki Murakami, Kiyoshi Suzuma, Masahiro Fujimoto, Yoko Dodo, Akitaka Tsujikawa

**Affiliations:** Department of Ophthalmology and Visual Sciences, Kyoto University Graduate School of Medicine, Kyoto, Japan

**Keywords:** Predictive markers, Retinal diseases

## Abstract

The early remission of diabetic macular edema (DME) often occurs in eyes treated with anti-vascular endothelial growth factor (VEGF) treatment. We retrospectively reviewed and characterized eyes with early remission of DME at six months in 80 eyes under pro re nata (PRN) intravitreal ranibizumab (IVR) injections. The number of eyes without center-involved DME gradually increased and 14 and 20 eyes achieved remission of DME at 3 or 6 months, respectively, under the PRN regimen following three monthly loading doses. In particular, eyes with early remission at 6 months had smaller CSF thickness than those without the remission before and after the treatment except at the 1-month visit (*P* < 0.05); however, the changes in CSF thickness did not differ between them. VA and its changes were not different between eyes with and without remission. Multivariate analysis revealed that smaller CSF thickness at baseline predicted the early remission of DME under PRN IVR injections (odds ratio, 0.989; 95% confidence interval, 0.982–0.997; *P* = 0.008). These data elucidate the clinical characteristics of early remission of DME under PRN IVR injections and suggest that smaller CSF thickness at baseline is a novel predictor of early remission under PRN IVR injections for DME.

## Introduction

Diabetic macular edema (DME), a vision-threatening diabetic retinopathy (DR), is characterized by the breakdown of the blood-retinal barrier (BRB) and concomitant neuroglial dysfunction^1,2^. Basic researches have revealed that vascular endothelial growth factor (VEGF) plays a pivotal role in the pathogenesis of DME, and clinical studies have demonstrated that anti-VEGF treatment has beneficial effects in DME patients^3–6^. Two major biological drugs against VEGF, ranibizumab and aflibercept, have been used as first-line therapies among several lines of therapeutic strategies since the approval by administrations^6–10^.

Despite the anatomical and functional efficacy, clinicians have to consider the rare but severe adverse effects of anti-VEGF treatment, e.g., endophthalmitis and lethal arteriothrombotic diseases^11^. Another issue is the socioeconomic burden for DME patients and communities^12–14^. Anti-VEGF management requires a much higher cost-per-quality-adjusted life years than other conventional interventions for DME. These concerns encourage clinicians to select the pro re nata (PRN) regimen and to discontinue the injections after DME has become stable^5,15^. Significantly, the number of intravitreal ranibizumab (IVR) injections is dramatically reduced during the second year or later in DME patients treated according to the PRN regimen, despite chronic nature of this disease^16^. This suggests that anti-VEGF treatment can lead to the resolution or remission of DME in some cases, which would guarantee stable visual function.

The BRB breakdown has been evaluated by fluorescein dye leakage in classical fluorescein angiography (FA) images. Optical coherence tomography (OCT) noninvasively delineates morphologic changes in neuroglial tissues of the retina^17^. The central subfield (CSF) thickness measured by OCT is increased in DME and optical coherence tomographic hyperreflective foci represent extravasation in diabetic retinas^18,19^. Other morphological findings, e.g., cystoid macular edema (CME), serous retinal detachment (SRD), vitreoretinal abnormalities, and photoreceptor damage, suggest several kinds of neuroglial disturbances in DME^20–22^. Some OCT findings are prognostic factors after anti-VEGF treatment; however, it remains to be elucidated what can predict the remission of DME under anti-VEGF treatment according to PRN dosing^21,23^.

In this study, we evaluated early remission of DME under PRN IVR injections and investigated its association with systemic or ocular characteristics at baseline to find predictors for early remission.

## Results

### Remission of center-involved DME at 6 months under IVR PRN injections

After the exclusion of 11 eyes at baseline and 65 eyes that were lost to follow-up before the 12-month visit, we retrospectively reviewed 80 eyes of 74 patients who received IVR PRN injections for center-involved DME, The baseline characteristics are shown in Table 1. Logarithm of the minimum angle of resolution visual acuity (logMAR VA) significantly improved from 0.260 (interquartile range [IQR], 0.155–0.523) to 0.155 (0.046–0.301), and CSF thickness decreased from 445 μm (392–546) to 300 μm (270–379) at 12 months in all 80 eyes (*P* < 0.001 in both comparisons, Supplementary Table). The number of IVR injections was 6 (4–8) during the 12 months.

**Table 1:** Baseline Characteristics.

Parameter	
Eyes/patients	80/74
Age (years)	69 (60–73)
Men/women	41/33
Hemoglobin A1c (%)	7.1 (6.7–7.8)
Systemic hypertension (patients)	45
LogMAR VA	0.260 (0.155–0.523)
International classification	
Mild NPDR	1 eye
Moderate NPDR	47 eyes
Severe NPDR	15 eyes
PDR	17 eyes
Pseudophakia	28 eyes
Panretinal photocoagulation	50 eyes
CSF thickness (μm)	445 (393–545)
Cystoid abnormalities	67 eyes (Kappa coefficient = 1.000)
Subretinal fluid	19 eyes (Kappa coefficient = 1.000)
Vitreoretinal abnormalities	5 eyes (Kappa coefficient = 0.882)
Disrupted EZ line (%)	9.6 (0.0–28.0) (ICC = 0.944)
Hyperreflective foci in the inner retinal layers	65 eyes (Kappa coefficient = 0.843)
Hyperreflective foci in the outer retinal layers	43 eyes (Kappa coefficient = 0.875)

**Figure 1:**
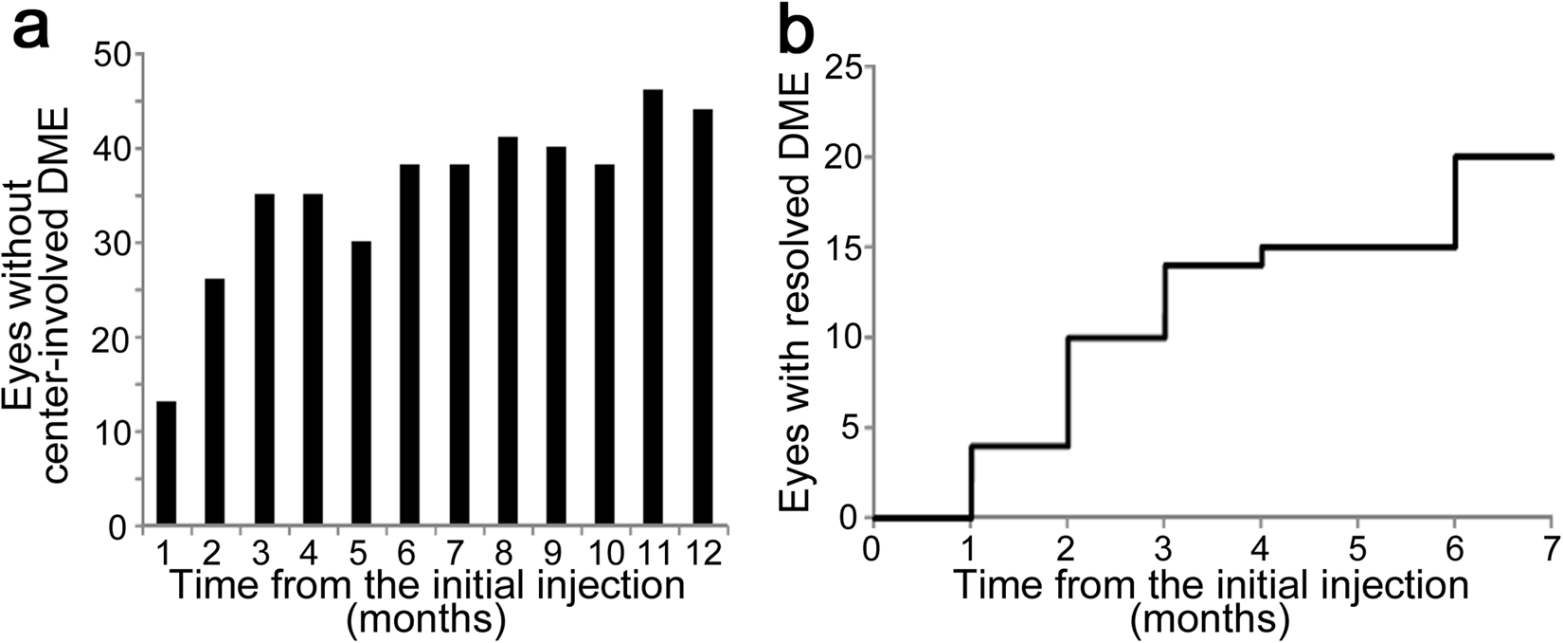
The number of cases with early remission of DME following PRN IVR injections. (**a**) The number of eyes without center-involved DME treated with PRN IVR injections following three monthly doses. (**b**) Cumulative cases with early remission at individual time points.

We counted eyes without center-involved DME at each time point, and found that such eyes gradually increased (Fig. 1a). The CSF thickness was below the threshold in 13, 34, 37, 40, and 43 eyes at 1, 3, 6, 9, and 12 months, respectively. Some eyes received PRN IVR retreatment at the recurrence of center-involved DME, whereas center-involved DME was not observed from the 1-month to the 12-month visit in 4 eyes (Fig. 1b). Fourteen and twenty eyes had DME remission at 3 and 6 months and received 4 (3–7) and 4.5 (3.25–7) IVR injections during the 12 months, respectively.

**Figure 2:**
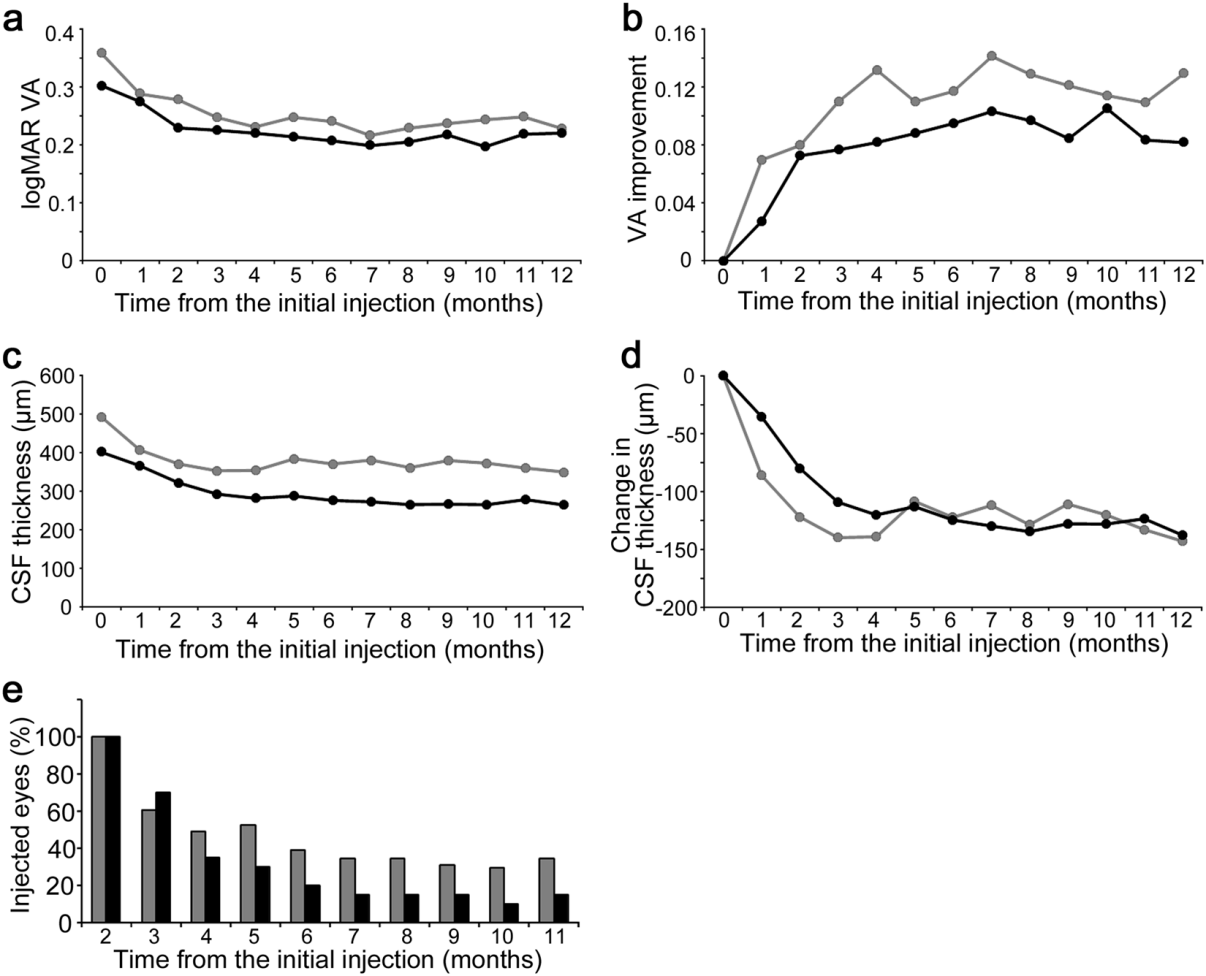
Comparisons in functional and anatomical courses between eyes with and without early remission of DME at 6 months following PRN IVR injections. The course of the logMAR VA (**a**) and its improvement (**b**) in eyes with and without early remission of DME under PRN IVR injections following three monthly loading doses. The course of the CSF thickness (**c**) and its changes (**d**). (**e**) The percentage of eyes that received IVR injection at individual time points. Black = eyes with early remission of DME at 6 months. Gray = eyes without early remission.

Comparative studies showed that logMAR VA and its changes were not different between eyes with and without DME remission at 6 months (Fig. 2a,b). Eyes with DME remission at 6 months had a smaller CSF thickness than those without DME remission at all time points except at the 1-month visit (*P* < 0.05; Fig. 2c). In contrast, the changes in CSF thicknesses did not differ between eyes with and without remission (Fig. 2d). Only two to four (10.0 or 20.0%) of the 20 eyes with early remission received IVR injection at the 6-month visit or later, whereas ranibizumab was administered in 18 to 24 (30.0 to 40.0%) of 60 eyes without early remission (Fig. 2e).

### Predictors of DME remission at 6 months

We applied logistic regression analyses to determine the baseline parameters to predict DME remission at 3 or 6 months under PRN IVR injections. According to the univariate logistic regression analyses, only smaller CSF thickness (odds ratio [OR], 0.990 [95% confidence interval {CI}, 0.982–0.998]; *P* = 0.015) at baseline were associated with DME remission at 3 months. Similarly, univariate analyses revealed that preoperative CSF thickness and hyperreflective foci in the inner retinal layers were negatively associated with DME remission at 6 months (Table 2). Further multivariate analyses demonstrated that only a smaller CSF thickness at baseline was correlated with DME remission at 6 months (Table 2).

**Table 2:** Multivariate Logistic Regression Analysis to Determine the Predictor of Diabetic Macular Edema (DME) Remission at 6-Month Visit or Earlier.

Parameter	Univariate		multivariate	
	OR (95% CI)	*P*-value	OR (95% CI)	*P*-value
Age (years)	1.063 (1.000–1.130)	0.051	—	—
Gender (Female)	0.678 (0.238–1.935)	0.468	—	—
Hemoglobin A1c (%)	0.835 (0.528–1.321)	0.442	—	—
Systemic hypertension	0.689 (0.248–1.920)	0.477	—	—
LogMAR VA	0.433 (0.055–3.379)	0.424	—	—
PDR	0.341 (0.071–1.642)	0.180	—	—
Pseudophakia	0.963 (0.355–2.960)	0.963	—	—
Panretinal photocoagulation	0.525 (0.189–1.461)	0.217	—	—
CSF thickness (μm)	0.988 (0.981–0.996)	0.003	0.989 (0.982–0.997)	0.008
Cystoid abnormalities	1.111 (0.273–4.515)	0.883	—	—
Subretinal fluid	0.496 (0.128–1.921)	0.310	—	—
Vitreoretinal abnormalities	0.000 (0.000–1.798 × 10^308^)	0.999	—	—
Disrupted EZ line (%)	1.000 (0.998–1.002)	0.934	—	—
Hyperreflective foci in the inner retinal layers	0.280 (0.086–0.914)	0.035	0.468 (0.123–1.785)	0.266
Hyperreflective foci in the outer retinal layers	0.374 (0.131–1.070)	0.067	—	—

## Discussion

In this study, we compared the functional and anatomical courses in eyes with and without early remission of DME and investigated the baseline parameters that predict early remission of DME under PRN IVR injections. Although DME is generally known as a chronic disease, 20 (25.0%) of 80 eyes achieved early remission under PRN dosing of this anti-VEGF drug with transient effects. Although visual prognosis or efficacy did not differ between eyes with and without early remission of DME, eyes with early remission had smaller CSF thickness at most time points than those without remission. In particular, multivariate analysis revealed that smaller CSF thickness at baseline is a predictor of DME remission at 6 months or earlier under PRN IVR treatment. This study therefore might propose early remission of DME as a possible benefit of patients who receive PRN IVR injections, in addition to visual improvement and a smaller number of injections.

Among several treatment strategies for DME, anti-VEGF treatment is the first-line in terms of great efficacy; however we have to consider the balance between medical benefit and the socioeconomic burdens^5,12–14^. Because eyes with a smaller CSF thickness at baseline were more likely to achieve early remission than those with a greater CSF thickness, we may consider that such cases are suitable for anti-VEGF treatment. The fixed or treat-and-extend regimens over a longer period may lead to an overdose and eventually a higher frequency of complications in such cases^6,7,24,25^. In contrast, eyes with a greater CSF thickness may not reach remission until 6 months and often relapse under PRN dosing. We might select other treatment regimens, e.g., PRN injections with more loading doses, fixed or treat-and-extend regimens, to achieve remission in such cases. Several inflammatory cytokines are increased in the intraocular humor of DME patients and basic research supports that neuroinflammation promotes the pathogenesis of DME^3,26,27^. Such mechanisms cannot be completely reversed by anti-VEGF treatment, and the combination therapy with other interventions, e.g., steroids or other anti-inflammatory drugs, might increase the frequency of early remission and reduce socioeconomic burdens^5,10,28^. Further prospective and comparative studies should be planned to elucidate the best strategies for DME eyes with a greater CSF thickness.

Visual outcomes or improvement did not differ between eyes with and without early remission of DME in this study. This is, to some extent, consistent with the reports from DRCR.net showing the discrepancies between functional and anatomical changes after interventions in some patients with DME^29^. Post hoc analyses of clinical trials have elucidated that poor VA, SRD, and no vitreoretinal abnormalities predict greater visual gain after anti-VEGF treatment, whereas the current study showed that these factors are not predictors of early remission of DME^21,23^. The improved photoreceptor status at the fovea was related to VA improvement under PRN IVR treatment, and photoreceptor damage did not differ between eyes with and without early remission (data not shown)^30,31^. Thus, early remission of DME might be an important aim of treatment; however this was not necessarily related to improvement in visual function.

It remains to be elucidated why a smaller CSF thickness was a predictor of early remission of DME following PRN IVR injections. Although both hyperreflective foci and retinal thickening are representatives of vascular hyperpermeability, multivariate analyses suggested that hyperreflective foci were a confounding factor in the prediction of early remission^19,32^. Retinal thickening may also be regulated by other factors, e.g., the loss of structural integrity. Sectional OCT images depict the retinal thickening are composed of CME, SRD, and sponge-like swelling^20^. We observed CME rather than SRD in persistent or recurrent DME at later time points (data not shown). CME is associated with ischemia and enlarged cystoid spaces may represent the degeneration of Müller cells and the concomitant loss of structural integrity^22,33,34^. We speculate that ischemic or degenerative changes in neuroglial tissues allow the accumulation of extracellular fluids under the minimal BRB breakdown and anti-VEGF treatment could not completely reverse the retinal thickening in such cases^35^. Another possibility may be that the greater retinal thickness depends on the combined mechanisms of VEGF and other cytokines, and the neutralization of VEGF alone could not block the pathophysiological mechanisms completely^3,26,27^.

A few eyes received IVR injections after DME remission, because VA or qualitative OCT findings were improved after DME remission according to the retreatment criteria of the Ranibizumab Monotherapy or Combined with Laser versus Laser Monotherapy for Diabetic Macular Edema (RESTORE) study. However, the eyes without early remission had a greater CSF thickness and received the injections more frequently at later time points, which is consistent with the association between retinal thickness and injection frequency according to the PRN regimen^36,37^. Although anti-VEGF treatment provides better functional efficacy, some patients with greater CSF thickness might consider switching to or combining other therapeutic strategies^15,25,38^.

This study with a retrospective nature had several potential limitations. We investigated the characteristics of early remission of DME which was defined during twelve months. Longer studies should be performed to elucidate whether DME remission is sustained or if retinal thickening relapses at the later time points and if present, the relapsed cases should be characterized. All participants were Asian and treatment was administered according to the PRN regimen in a single center. We converted decimal VA to logMAR VA, so the VA changes are approximate and not so sensitive. The meausurement of logMAR VA or Early Treatment Diabetic Retinopathy Study (ETDRS) chart may guarantee high accuracy in VA changes in the future study. The data were analyzed in a retrospective nature, so we could not completely exclude the selection bias. The number of eyes may be too small for multivariate analyses. Prospective multicenter studies with a large number of cases must evaluate the reproducibility in other races treated using other regimens for a longer period.

In conclusion, some patients with DME achieved early remission following PRN IVR injections, which might be predicted by a smaller CSF thickness at baseline. This suggests that the PRN IVR regimen for such cases may reduce the injection frequency; however other regimens or additional treatments may be necessary for eyes with a greater CSF thickness at baseline to achieve DME remission.

## Methods

### Participants

In this retrospective, interventional case series, we reviewed 80 consecutive eyes of 74 patients who received PRN IVR injections for center-involved DME for 12 months or longer. These patients were referred to Kyoto University Hospital from March 2014 to August 2017 for the baseline visit. All research and measurements were performed in accordance to and with adherence to the tenets of the Declaration of Helsinki and the study protocol was approved by the Kyoto University Graduate School and Faculty of Medicine, Ethics Committee. Written informed consent was obtained from all participants before study enrollment.

The inclusion criteria were adults ≥20 years with type 1 or 2 diabetes mellitus, visual impairment due to center-involved DME and not other causes, center-involved DME that was treated with IVR injections according to the 3+ PRN regimen for 12 months or longer, and a signed informed consent. The exclusion criteria at baseline were eyes with media opacity affecting the VA, other chorioretinal diseases, angiogenic complications (neovascular glaucoma, vitreous hemorrhage, or tractional retinal detachment), any intervention (anti-VEGF therapy, focal/grid photocoagulation, vitreoretinal surgery, and intraocular or periocular corticosteroids) for DME or retinal photocoagulation within the previous 6 months, previous intraocular surgery other than cataract extraction (vitrectomy and glaucoma surgery), cataract surgery within the previous 3 months. Further exclusion criteria were drop-out during the 12-month follow-up due to patient’s inconvenience or a patient’s desire to terminate treatment or switch to other therapeutic strategies for DME (aflibercept injection, focal/grid photocoagulation, vitrectomy, and intraocular or periocular corticosteroids). We excluded eyes with tachyphylaxis to ranibizumab injection or additional treatments (focal/grid photocoagulation, panretinal photocoagulation, vitrectomy [for vitreous hemorrhage], or cataract surgery) for DME based on doctor’s discretion for the 12 months.

### Intervention

Ranibizumab was intravitreally administered according to the 3+ PRN regimen described in the RESTORE study^15^. Ranibizumab (0.5 mg) was injected 3.5 mm posterior to the limbus after disinfection. Three monthly loading doses were followed by PRN injections at monthly visits according to the retreatment criteria of the RESTORE study.

### Optical coherence tomography

The best-corrected decimal VA was converted to logMAR VA, followed by a comprehensive ophthalmic examination. OCT images were acquired using spectral-domain (SD)-OCT (Spectralis OCT, Heidelberg Engineering, Heidelberg, Germany) at every monthly visit. The raster scan mode was applied to obtain three-dimensional images and to measure the CSF thickness of the ETDRS grid (the mean retinal thickness within a 1 mm circle centered on the fovea) as previously described^18,39^. Further, we obtained vertical and horizontal retinal sectional images dissecting the fovea using the 30-degree cross-hair mode and averaged 20 to 100 images to construct better images, as previously described^40^.

A few publications reported that the number of injections was dramatically reduced in the second year under PRN IVR injections for DME, despite its chronic clinical course^16^. Actually, clinicians often observe the sustained disappearance of center-involved DME after several IVR injections. This encouraged us to hypothesize that the remission of center-involved DME occurs at earlier time points in some cases. In this study, eyes without center-involved DME from any time point to the 12-month visit were defined as in DME remission at the time point. For example, eyes without center-involved DME from the 3-month to the 12-month visit were referred to as ‘DME remission at 3 months’.

We further assessed several parameters within the central 1 mm in both vertical and horizontal sections. Three OCT findings, i.e., cystoid abnormalities, subretinal fluid, and vitreoretinal abnormality, were qualitatively evaluated as previously described^21^. The foveal photoreceptor damage was manually quantified as the transverse length of the disrupted ellipsoid zone of photoreceptors (EZ) line within the central 1 mm^22^. We defined hyperreflective foci between the external limiting membrane (ELM) and the surface of the retinal pigment epithelium as those in the outer retinal layers, as previously described^41^. Hyperreflective foci between the inner limiting membrane and the inner surface of the ELM were referred to as being in the inner retinal layers^19^. Two retinal specialists assessed these OCT parameters independently. They had a discussion at the disagreements in the qualitative parameters. The average of the quantitative parameters was applied for further analyses.

We evaluated the association between systemic and ocular parameters at baseline and early remission of DME under PRN IVR injections as a main outcome measure.

### Statistics

The results are expressed as the median (IQR). After test of normal distribution using the Shapiro-Wilk test, the comparative nonparametric data were analyzed using the Mann-Whitney U-test. Significant differences in the sampling distributions were evaluated using Fisher’s exact test. The Kappa coefficient or intraclass correlation coefficient (ICC) were used to evaluate the concordance in the qualitative or quantitative parameters, respectively. Univariate and multivariate logistic regression analyses were employed to evaluate the ability of systemic or ocular parameters at baseline (independent variables: age, gender, HbA1c, systemic hypertension, logMAR VA, proliferative diabetic retinopathy [PDR], pseudophakia, panretinal photocoagulation, CSF thickness, cystoid abnormalities, subretinal fluid, vitreoretinal abnormalities, disrupted EZ line, and hyperreflective foci in the inner or outer retinal layers) to predict DME remission at 3 or 6 months under PRN IVR injections. Significant factors in univariate analyses (*P* < 0.05) were selected and applied to multivariate logistic regression analysis. In the preliminary 40 eyes, DME was remitted at 6 months in 10 eyes and two independent variables (CSF thickness and hyperreflective foci in the inner retinal layers) were associated with the remission (*P* < 0.05) according to univariate logistic regression analyses. A sample size of 20 or more eyes with DME remission (or 80 or more eyes with or without the remission) is required for the multivariate analysis, as described previously^42^. *P* < 0.05 was considered statistically significant. SPSS version 24.0 was used for statistical analyses (SPSS, Inc., Chicago, IL, USA).

## Supplementary information

Supplementary Table
