# Supplementary material for: Predictor of Early Remission of Diabetic Macular Edema under As-Needed Intravitreal Ranibizumab
Source: Sci Rep. 2019 May 20;9:7599. doi: 10.1038/s41598-019-44078-6 (PMC6527559; doi:10.1038/s41598-019-44078-6)
Supplement: Supplementary file 1 — Supplementary Table [file 41598_2019_44078_MOESM1_ESM.pdf]

Supplementary Information

Predictor of Early Remission of Diabetic Macular Edema under As-Needed Intravitreal Ranibizumab

Tatsuya Yoshitake, MD; Tomoaki Murakami, MD, PhD; Kiyoshi Suzuma, MD, PhD; Masahiro Fujimoto, MD; Yoko Dodo, MD; Akitaka Tsujikawa, MD, PhD.

Department of Ophthalmology and Visual Sciences, Kyoto University Graduate School of Medicine, Kyoto, Japan.

Correspondence and requests for materials should be addressed to Tomoaki Murakami, MD, Department of Ophthalmology and Visual Sciences, Kyoto University Graduate School of Medicine, 54 Shogoin-Kawaracho, Sakyo, Kyoto 606-8507, Japan (phone: 81-75-751-3250; fax: 81-75-752-0933; email: mutomo@kuhp.kyoto-u.ac.jp)

E-mail: mutomo@kuhp.kyoto-u.ac.jp.

TABLE. Shapiro-Wilk test for normality.

Parameter	Statistic	Significance
Age (years)	0.901	<0.001
Hemoglobin A1c (%)	0.885	<0.001
LogMAR VA	0.861	<0.001
CSF thickness (μm)	0.952	0.013
Disrupted EZ line (%)	0.749	<0.001
